# The Importance of Genuineness in Public Engagement—An Exploratory Study of Pediatric Communication on Social Media in China

**DOI:** 10.3390/ijerph17197078

**Published:** 2020-09-27

**Authors:** Wenze Lu, Cindy Sing Bik Ngai, Lu Yang

**Affiliations:** Department of Chinese and Bilingual Studies, The Hong Kong Polytechnic University, Hong Kong SAR HK, China; wen-ze.lu@connect.polyu.hk (W.L.); jasminelu.yang@connect.polyu.hk (L.Y.)

**Keywords:** pediatrics, social media, online communication, genuineness, public engagement

## Abstract

There is a growing need for the public to interact with pediatricians through social media in China, and genuineness is a crucial factor contributing to effective communication, but few studies have examined the relationship between genuineness and its effect on public engagement. This study developed a four-dimension framework including self-disclosure, genuine response, functional interactivity, and genuineness in Chinese culture to investigate the effect of genuineness in the communication of Chinese social media influencers in pediatrics on public engagement. Content analysis was employed to examine these dimensions and the related public engagement in 300 social media posts on the largest microblogging site in China. The findings indicate that genuine response was positively associated with the number of comments and positive comments, while negatively related to the number of shares. Functional interactivity made the site more appealing, resulting in likes and shares. Genuineness in Chinese culture was reflected in engagement through sharing posts by the public. This study is the first to develop an integrated framework to measure genuineness in online health communication and contributes to the understanding of the effect of genuineness on Chinese public engagement in social media.

## 1. Introduction

Child health and development has been one of the biggest issues in the World Health Organization. In recent years, Chinese president Xi Jinping has put public health at the center of the country’s policy-making agenda, clarifying the need to include public health in official government policy. “The Healthy China 2030 Planning Outline”, issued by the Chinese State Council, is the first long-term strategic plan of public health developed at the national level in China [1]. One of the aims in the plan is to enhance children’s health and reduce children’s mortality by the construction of pediatrics and the prevention of pediatric critical diseases [2]. As an important area of study in public health, children health advocates the prioritization of children healthcare in public health community as a basis for the improvement of national health [3]. Concerning this great emphasis on children’s health, sustainable investment and efforts have been put into relevant fields, especially online.

Chinese Premier Li Keqiang once put forward a guideline named “Internet+,” aiming to integrate online resources with other domains including education, logistics, and health care. Social media is of particular importance in disseminating health information and promoting health communication as China records the world’s largest number of registered social media users [4,5,6]. Of all the social media in China, Sina-microblog (aka Weibo) is one of the most popular platforms for health communication, with 516 million active online users at the end of 2019 [4]. In recent years, the number of doctors’ accounts in Weibo has been booming. It is estimated that Weibo has more than 50,000 registered doctors and generated 70 million followers in total, which facilitates the dissemination of high-quality health information [7].

These online doctors are coined as social media influencers (SMI) in our study. SMI refers to those who are well connected with social network users and persuasive in the sphere in which they exert opinion leadership to affect users’ decision-making process [8,9]. They play a crucial role in forming public views and impacting the spread of information in social media [8]. It is their expertise, ease of access, and social ties on social media platforms that enable them to influence followers [9]. In this regard, social media influencers in pediatrics (SMIP) are more convenient for consultations, as they use social media to address parents’ concerns. This includes opening online clinics, spreading children’s health knowledge online, responding to questions online, sharing thoughts of children’s health issues in blogs, and more. Likewise, owing to their expertise and the ease of access for their followers, SMIP play an essential role in the circulation of children’s health knowledge, formation of public opinion, and the behavioral effect of online followers [10]. Given that acquiring children healthcare information from, interacting with, and following SMIP online is becoming more commonplace, attention should be paid to the quality of health information and the effectiveness of SMIP’s health communication.

### 1.1. Previous Studies on Health Communication via Chinese Social Media

In recent years, health communication via social media has received extensive attention from international academic communities and health practitioners. Moorhead et al. [6] reviewed 98 worldwide research studies to identify the benefits, limitations, and uses of social media (e.g., Twitter, Facebook) for health communication among the general public, which provides insights for future health communication research. Given that social media have been widely used in China, scholars began to place more emphasis on health communication with the public via Chinese social media. Previous studies have identified possible factors affecting online health communication in China. These studies mainly focus on the analysis of non-content factors. For instance, Gao et al. [11] argued that Chinese users’ perceived credibility of health information related to participants’ involvement level and prior knowledge. Jiang [12] illustrated that the presence of interactive functions in Chinese social media contributed to productive communication between health care organizations and the public. Moreover, prior study found that social networks and social support affected how Chinese people viewed the health issue and disseminated specific information [13]. A few studies looked into the content of health information on social media. Jiang and Beaudoin [14] studied the smoking information posted from an organization’s account on Weibo. The results uncovered that perceived risk and self-efficacy content positively influenced audience engagement. Liu et al. [15] illustrated that messages emphasizing the benefits of recommended healthy practices and harmful outcomes of unhealthy practices were associated with a Chinese user’s behavior, such as shares and comments. Tian et al. [16] analyzed online messages indicating depression, which provided insights for medical practitioners to better communicate with people suffering from depression in China.

To sum up, we found that most of the studies fell into the following three categories: (1) the effectiveness of health information provided by Chinese organizations and non-professional online users [13]; (2) typical health problems, such as depression, smoking addiction, and HIV in China [16]; (3) positive effects of Chinese social media on health communication [6]. However, few empirical studies focused on health information provided by Chinese doctors via social media, especially pediatricians. Likewise, the effectiveness of driving factors in Chinese SMIP’s communication on public engagement has not been sufficiently discussed. Public engagement is important because it reflects the public’s attitude and affects their trust and relationship with involved members [17,18]. When there is a high level of public engagement, the public’s panic, anxiety, and uncertainty will be minimized and the trust on the SMI will be improved [19,20]. Therefore, the study of the effectiveness of driving factors in Chinese SMIP’s online communication on public engagement is highly warranted. The study can help pediatricians, and other doctors, better understand the constituent parts of online messages to be employed when they attempt to engage the public in health conversations through social media. Moreover, a better understanding of how people view doctors’ online health communication would strengthen the value of the principles that guide good communication.

### 1.2. The Importance of Genuineness and Its Role in Health Communication

Among all the driving factors in health communication, genuineness has been suggested as a contributing factor in effective medical communication, especially in patient-centered psychotherapy [21]. Norcross and Newman [22] pointed out that health practitioners considered doctors’ genuineness as “important for significant progress in psychotherapy, and, in fact, fundamental in the formation of a working alliance” (p. 104). Likewise, therapists’ characteristics, especially genuineness, authenticity and honesty can enhance their credibility which was essential for promoting therapeutic alliance and patients’ trust [23,24,25]. Genuineness has been widely studied in face-to-face communication between doctors and patients. Little attention has been paid on the importance of genuineness on health communication in the context of social media. Due to the absence of concrete operational dimensions in studying genuineness, we developed an integrated framework that included four dimensions-- “self-disclosure”, “genuine response”, “functional interactivity” and “genuineness in Chinese culture”, for examining genuineness in social media communication based on previous studies in health communication, dialogic communication, and the study of Chinese culture. The first three are universal dimensions that occur regardless of the cultural context while the fourth dimension is a cultural determined dimension which is essential to Chinese communication.

#### 1.2.1. Self-Disclosure

There is no universally agreed-upon definition of genuineness. However, the common features of genuineness focus on “self-dimension,” referring to transparency, realness, and the authenticity of one’s mind and behavior. In the medical field, Landreth stated, “the most significant resource the therapist brings to therapy relationship is the dimension of self. Skills and techniques are useful tools, but therapist’s use of their personalities is their greatest asset” ([26], pp. 104–105). Egan also specified that genuineness is “beyond professionalism and phoniness” ([27], p. 55). It refers to an attitude or behavior that can only be expressed if the doctor is self-aware [28]. Similarly, studies have noted that doctors’ genuineness could be conceptualized as being real, being their true authentic self, and getting rid of dishonest and false behavior [29]. Nevertheless, how to concretely perceive genuineness via “self” has been understudied. Previous studies once demystified the idea that “self-dimension” of doctors’ genuineness could be identified by self-disclosure during the health care process [30,31,32].

Self-disclosure is defined as being willing to consciously and intentionally reveal personal feelings, life experiences, and professional knowledge in the process of communication to establish a positive relationship [30]. Self-disclosure has received extensive attention in medical research because of its benefits to patients’ positive health practices and doctor-patient relationships [31]. Previous research [30,31,32] found three main types of self-disclosure being preferred by the doctors, namely the disclosure of personal thoughts/feelings, disclosure of personal life, and disclosure of personal expertise (e.g., pediatrics, neurology and psychiatry). A study reported [31] that patients liked their doctors more when doctors disclose personal feelings and thoughts. Patients viewed an act of expressing feelings and thoughts from doctors as friendly and helpful because it encouraged patients to participate in a dialogue and enhance patient’s self-exploration [31]. Another study suggested that when a doctor disclosed his/her own lifestyle (e.g., positive health behaviors or daily activities), patients considered the doctor to be more credible and approachable [32]. Likewise, patients particularly valued when doctors disclosed the accumulated skills, experience, and specific expertise in the field [28,29,30]. Expanded on the previous studies, our study aims at investigating these three types of self-disclosure exhibited in the SMIP communication, and how public responded to different types of disclosure.

#### 1.2.2. Genuine Response

In addition to self-disclosure, prior studies [33,34,35,36] confirmed that genuineness could be manifested when healthcare workers communicate consistently and provide expertise and emotional support to patients. In the health communication, a consistent response from doctor matters because it reflects “the degree to which one person is functionally integrated in the context of the relationship with another, such that there is an absence of conflict or inconsistency between their total experience, their awareness, and their overt communication in their congruence in the relationship” ([34], p. 12). A genuine response is not a response that simply expressed Yes or No answer or a simple act of reaction (e.g., smile/cry). It emphasizes on the recognition of interlocutors’ concerns, thereby providing professional and emotional support to address their problems [33,34,35,36]. A genuine response to a patient’s question or concern, is useful for building a positive therapeutic relationship [35]. 

Yet, an absence of an analytical framework for examining genuine response was noted. As such, we have modified frameworks from previous studies [33,36] on health communication studies and proposed three main sub-dimensions to measure genuine response: (1) consistency, (2) knowledge, skill, experience and treatment advice, and (3) facilitation of hopefulness. Consistency emphasizes on whether patients’ concerns are well understood, and the response is on the right track [33,36]. Van et al. [36] noted that healthcare workers often rephrase or repeat the patient’s questions or concerns before providing follow-up treatment and explanation to demonstrate their understanding on patients’ needs. Bottorff et al. [33] pointed out that nurses who responded with expert knowledge, such as treatment and medical advice were able to reduce patients’ anxiety and uncertainty. They suggested that such responses enable patients to make informed decision-making and be more actively involved in a dialogue [33]. Moreover, previous research [33,35,36] indicated that nurses usually communicated emotional support through facilitation of hopefulness with patients during a therapeutic process. Bottorff et al. [33] and Van et al. [36] found that facilitation of hopefulness that nurses adopted in interactions contributed to reassuring patients and avoiding escalation of emotional instability, thereby leading to positive outcomes of treatment. In view of these, we intend to investigate and reveal genuine responses in SMIP messages by examining the three sub-dimensions adapted from previous studies on health communication [33,36].

#### 1.2.3. Functional Interactivity

In addition to health communication studies, this study drew on insights from dialogic communication theory in public relation and communication studies where functional interactivity serves as one of the principle elements in creating a genuine and dialogic communication online [37]. Functional interactivity refers to the interface’s elements that allow an online user to interact with someone/an organization and build a dialogue between interlocutors [38]. Such elements include hyperlinks, multimedia, live-chat rooms, and questions [39]. Functional interactivity is of particular importance in social media communication where dynamic, two-way interactive communication is advanced by the proliferation of social media [40,41]. 

For a genuine and dialogic communication to emerge [37,40], interactive functions including “generation of return visits,” “conservation of visitors,” and “dialogic loop” were deemed necessary. The “generation of return visits” emphasizes on the return visit of the public while the “conservation of visitors” highlights the importance of connecting the public to the SMI. Both “generation of return visits” and “conservation of visitors” could be achieved by providing external links and hashtags to engage the public [41,42]. “Dialogic loop” placed much attention on promoting dialogue between SMI and the public where strategies including providing frequent responses, asking questions, and using multimedia are most employed [41,42]. Subsequently, this study examines the use of interactive functions for building genuine dialogue in SMIP communication and their association with public engagement.

#### 1.2.4. Genuineness in Chinese Culture

If the first three dimensions of genuineness are universal dimensions that occur regardless of the cultural context, the fourth dimension can be identified as a cultural determined aspect which is essential in Chinese communication. In Chinese culture, honesty and kindness are viewed as necessary components for developing genuine dialogue [43,44,45,46], and therefore, are of particular value to the Chinese audience. 

Honesty is the essence of Confucianism and has a deep impact on the moral personality development [47]. Kindness, along with compassion, care, friendliness, righteousness, and affection, is one of the Confucian values about a “good person” in Chinese culture [48]. A kind individual is positively related to excellent job performance [49]. Zhang et al. [50] also illustrated that the kinder a nurse is, the more satisfied patients are.

The genuineness in SMIP communication that attributes to the portrayal of a positive personality trait [46,47], could be measured by the use of lexical indicators for the expression of honesty and kindness [43,44,45,46]. Honesty in the Chinese culture is denoted as the moral quality of being consistent in words and deeds; opposite to hypocrisy; loyalty and open-mindedness; no lying, no fraud, no exaggeration, no distortion of facts [51]. As such, we postulate that lexical indicators related to (1) reasoning and explanation (e.g., because, so), (2) personal sharing and views (e.g., I think, I contend, I prefer), and (3) truth/facts (e.g., In fact, the truth is, the evidence reveals) are important in expressing honesty. Kindness in the Chinese culture denotes personalities of being friendly, harmonious, kind-hearted and nice, and behaviors of altruistic, affectionate, righteous, and caring [52,53]. In SMIP communication, we expected kindness to be expressed through the use of lexical indicators related to (1) caring (e.g., is that okay, are you satisfied, is this clear for you), (2) friendliness (e.g., hello, could you please, welcome), (3) gratitude (e.g., thanks, appreciate it), (4) blessing (e.g., wish you, no worries, everything will be fine), and (5) compliment (e.g., good question, you are right).

### 1.3. Public Engagement in Social Media

In our study, public engagement refers to the public’s responses to the content communicated via social media which reflects the public’s cognition and attitude on a particular issue [54]. Different level of public engagement on social media reveals their trust and relationship with involved members [20,55]. Previous research has studied public engagement in different contexts with varied definitions. In corporate-stakeholder communication, Bruce and Shelley defined public engagement as “the interaction between an organization and those individuals and groups that are impacted by, or influence, the organization” ([56], p. 30). In CEO communication, Men et al. conceptualize public engagement “as a behavioral construct focusing on publics’ interactions with CEOs” ([57], p. 87). In government communication, public engagement refers to the involvement of citizens in public affairs [58]. In this aspect, public engagement aims to boost mutual understanding and build up a good relationship between the local government and the public [58].

In recent years, scholars have started to study public engagement and perception in an online context due to the arrival of global social media platforms [59,60], such as Weibo, YouTube, Twitter, and Facebook, which all include the common feature of real-time public interaction. Social media includes a variety of functions to engage with the public (e.g., blogs, photo sharing, video sharing, live chatting, and co-generation of content), and offers the ability to express attitudes via reaction buttons, appearing at the bottom of the relevant content: Like, Share, and Comment [60]. “Like” is an indicator to express awareness and interest, which can be used to identify the popularity of messages [61,62]. “Share” provides the opportunity to connect the organizational message to one’s social group, and “Comment” enables direct dialogue with organizations [61]. These engagement indicators fall into different engagement levels. Like is the lowest level of engagement as it requires less cognitive effort and commitment than other indicators [62]. Share has a higher engagement level [63], as it can be viewed not only as an important indicator of user recognition but as user recommendation. This indicates that sharing requires certain time to evaluate the post’s value [64]. A comment is the highest level of public engagement, as it requires more effort by the public to figure out the meaning of posts and directly respond to the messages with words or descriptors [61]. The number of likes and shares may indicate an overall positive effect but analyzing constituting parts embedded in comments helps estimate outcomes more concretely and accurately [65]. For instance, Fan [66] argued that how people perceive products could be revealed in the comments thread. By studying the comments, the organization will know the weaknesses and affordances of products. Public perceptions towards the content can also be amplified or constricted by reviewing other users’ comments [67]. Therefore, comments can be quite persuasive on affecting public opinions [68]. The present research categorizes comments as the high, shares as the intermediate, and likes as the low level of public engagement indicators.

Beyond the three engagement indicators, we paid particular attention to the valence of positive user comments. Kim and Yang [63] found that positive comments towards an organization are more likely to affect how people remember the organization, and further influence the organization’s reputation. In tourism, for example, positive e-comments on businesses strongly influence travelers who read e-comments when they decide to select a hotel [69]. In the health field, positive online comments were positively associated with the effectiveness of anti-smoking persuasion on the public’s attitudes [70].

### 1.4. Development of Research Questions

Given the impact of SMIP on public views and boosting children’s health, we aim to identify the effectiveness of genuineness, one of the most influential driving factors in health communication, in SMIP’s online communication. The paper employs the coding framework of four genuineness dimensions generated from dialogic communication, health communication and Chinese cultural studies to examine the association between genuineness and public engagement. Further, it provides an in-depth understanding on the relationships between genuineness and public reception indicators (i.e., likes, shares, comments, and positive comments).

For the first research question (RQ), we aim to investigate the association between the four dimensions of genuineness and public engagement, therefore the following research question is put forward:

RQ1: What is the association between the four dimensions of genuineness (“self-disclosure”, “genuine response”, “functional interactivity” and “genuineness in Chinese culture”) and public engagement?

To fully understand the relationship within sub-dimensions in each dimension and public engagement, our second set of research questions is formulated as follows:

RQ2a: What are the associations within the sub-dimensions of “self-disclosure” (“disclosure of personal life”, “disclosure of personal thoughts and feelings”, and “disclosure of personal expertise in pediatrics”) and public engagement?
RQ2b: What are the associations within the sub-dimensions of “genuine response” (“consistency”, “knowledge, skill, experience and treatment advice”, and “facilitation of hopefulness”) and public engagement?
RQ2c: What are the associations within the sub-dimensions of “functional interactivity” (“the generation of return visits and conservation of visitors”, and “dialogic loop”) and public engagement?
RQ2d: What are the associations within the sub-dimensions of “genuineness in Chinese culture” (“honesty” and “kindness”) and public engagement?

## 2. Materials and Methods

### 2.1. Sample Selection

First, we employed a self-developed Python program programmed by our research assistant with a postgraduate degree in computational science to identify the top pediatricians based on their number of followers in Weibo, one of the largest microblogging sites in China. The crawler is designed to search and identify verified pediatrician using the keywords “pediatrician” and the label “V-users”. “V-users” referred to verified users where doctors need to submit their medical certificates to Weibo to prove their authenticity. Once approved, the letter “V” with a yellow badge will be assigned to these doctors’ profile pictures. Verified pediatricians are preferred in our study, as they are much more influential in the social media community than non-verified ones [71]. The identified pediatricians with the highest number of followers in March are recognized as social media influencers in pediatric (SMIP) in our study as they are well connected and persuasive in their field.

### 2.2. Data Collection

As an exploratory study, we scrutinized the number of posts published by these top 10 SMIP for six months (from March 1 to August 31, 2019), to ensure that they are active communicators online. Subsequently, we replaced two inactive users who published fewer than two posts/day on average with the next two SMIP on the list. Table 1 presents the final list of Top 10 SMIP.

Unlike Twitter, Weibo tends to change its open API at times for the purpose of data security and timely technical updates. Moreover, Weibo has a strict “restrictions on the API usage rate and unsolicited data requests” ([72], p. 597). Therefore, we had to manually collect the SMIP posts, record the number of comments, likes and shares and analyze positive comments for our study. Due to the complexity of the ten sub-dimensions embedded in the four dimensions of genuineness, we decided to code the sub-dimensions manually to ensure an accurate interpretation [54] on the use of genuineness in the SMIP posts. Taking all these into considerations, we decided to harvest a sample size of 300 posts to represent the target population. We have employed the sample size calculator developed by the Australian Statistics Bureau [73] to estimate a sample size of 300, giving a confidence level of 95%, a confident interval of 0.056, and standard error of 0.029. Through systematic random sampling, we randomly sampled 30 posts from each SMIP’s Weibo account between March 1 and August 31 in 2019 for content analysis.

### 2.3. Coding Scheme and Procedure

Content analysis was employed to examine the four dimensions of genuineness adopted in the 300 posts of the top 10 SMIP on Weibo. Content analysis is a widely employed method in the study of media communication [74] and can be applied to “virtually any form of linguistic communication to answer the classic questions of who says what to whom, why, how, and with what effect” ([75], p. 268). It is concerned with the context where the occurrences of words, signs, and sentences are examined to provide in-depth understanding [74,76]. Researchers could adapt and integrate framework from previous research for conducting coding in content analysis [74,76]. In this study, we have drawn insights from health communication, dialogic communication and Chinese cultural studies (see Section 1.2.1, Section 1.2.2, Section 1.2.3 and Section 1.2.4) to develop a four-dimension framework in genuine communication for pediatricians. A code book that includes the four dimensions of genuineness, the ten sub-dimensions, and descriptors to investigate the genuineness in SMIP’s communication has been developed (see Table 2). The coding procedure of each dimension is listed below:

For the dimension of “Self-disclosure”, we coded the pediatrician’s willingness to disclosure information related to his/her: (1) personal life, (2) personal feelings and thoughts, and (3) personal expertise in pediatrics [30,31,32] in the post on sentence basis.

For the dimension of “Genuine response”, we coded to reveal if the pediatrician demonstrates: (1) consistency, (2) knowledge, skill, experience and treatment advice, and (3) facilitation of hopefulness in his/her response in comment thread on sentence basis [33,34,35,36].

For the dimension of “Functional interactivity”, we coded the number of interactive elements (e.g., links, hashtag, multimedia, responses) used to facilitate: (1) “the generation of return visits and conservation of visitors,” and (2) creation of “dialogic loop”. Refs. [37,39,41,42] in the post and comment thread.

For the dimension of “Genuineness in Chinese culture”, we coded the number of lexical indicators that demonstrates pediatrician’s personality: of (1) honesty, and (2) kindness [43,45,46]; Refs. [50,51,52,53] in the post and comment thread. 

To ensure a high accuracy of analysis, a face-to-face meeting was held by the first author and the second author before the coding exercise. The authors identified the related descriptors, including lexical indicators and features in each dimension. Relevant examples were retrieved from the database collected to guide the coders in the process of coding. The first author and a well-trained research assistant who possesses a MA in communication conducted the coding in this study. Table 2 presents the four dimensions, ten sub-dimensions, and descriptors of the code book. The related examples extracted from the database could be found in Appendix A.

For the evaluation of public engagement, the number of shares, likes, comments, and positive comments were identified. Beyond three engagement indicators, we paid particular attention to the valence of positive user comments, as positive comments can contribute to the excellent reputation of social media influencers and enhance public trust [65,77]. Online positive comments are characterized by the expression of compliment and affirmation, admiration and gratitude, usefulness and goodness [78]. In the corpus of this study, the comments, such as “thank you doctor,” “beneficial advice,” “great,” and “feel the same way” were recorded.

### 2.4. Interrater Reliability

The coding was conducted by the first author, the primary coder, and a well-trained research assistant who possesses a MA in communication. To ensure inter-rater reliability on the coding of “self-disclosure”, “genuine response”, “functional interactivity”, “genuineness in Chinese culture”, and public engagement, the coders were highly trained on the coding scheme. Any disagreement between the two coders was discussed in the coding process until the agreement was achieved. The measure of interrater reliability was based on the co-coding of 60 posts from the top two SMIP (20% of the total number of posts studied). For all categories, the average agreement was higher than 0.95, and the average Cohen’s Kappa was greater than 0.9, indicating an almost perfect agreement [79]. Please refer to Table 3 for the interrater reliability of all categories.

### 2.5. Data Standardization and Statistical Analysis

Since the content in posts and responses in comment threads varied from two words to 140 words, all coded data have been standardized, especially the coding done on sentence basis. As such we have standardized the coding data of the sub-dimensions in “self-disclosure” and “genuine response” by dividing the number of sentences yielded in each sub-dimension in every post by the overall number of sentences in each post. As for the sub-dimensions of “functional interactivity” and “genuineness in Chinese culture”, we standardized the data by dividing the number of features harvested in each sub-dimension in every post by the total count of features in each post.

As likes, shares, comments, and positive comments are count outcomes, Poisson regression was employed for statistics analysis. However, we found overdispersion exhibited when testing for assumptions in Poisson regression. Then we decided to employ negative binomial regression to replace Poisson regression as suggested in previous research [80,81]. 

Negative binomial regression (NB2) fits various types of data arising in communication research [81], and “the negative binomial model is a more general model compared with the Poisson regression model that relaxes the strong assumption that the underlying rate of the outcome is the same for each included participant” [82] (p. 3). Moreover, negative binomial regression allows various information to be included [82]; it is appropriate for the data in this study, especially in the presence of overdispersion. Thus, RQ1 and RQ2 were examined via negative binomial regression in which likes, shares, comments, and positive comments were taken as dependent variables. For the examination of associations between the four dimensions and public engagement in RQ1, standardized data in the sub-dimensions were summed up in the related dimension. For instance, the data in “disclosure of personal life”, “disclosure of personal feelings and thoughts”, and “disclosure of personal expertise” were combined to form the “Self-disclosure”. 

As for RQ2, we used the standardized data in the sub-dimensions to examine if there was a significant association between sub-dimensions in each genuineness dimension and public engagement.

## 3. Results

In this section, we aim to reveal the association between the four dimensions of genuineness and public engagement and then identify different levels of impact of sub-dimensions in each genuineness dimension on public engagement. In response to RQ1, the NB2 findings indicated the number of “genuine response” was positively associated with the number of comments and positive comments, but negatively related to number of shares. For every extra sentence on “genuine response”, 1.344 times more comments were generated, a statistically significant result (*p* < 0.0001). Similarly, there was a 16% increase in the number of positive comments for each extra sentence on “genuine response” (*p* = 0.0001). Likewise, a positive association was found between the occurrence of “functional interactivity” and shares, whereas there was a negative correlation with comment and positive comments. A 19.8% increase in the number of shares is expected for every extra feature in “functional interactivity” found (*p* = 0.0001). In addition, the frequency of “genuineness in Chinese culture” was positively related to the number of shares. For every extra lexical indicator in “genuineness in Chinese culture”, 1.122 times more shares were generated (*p* = 0.003). Table 4 summarizes the negative binomial regression results on the four dimensions of genuineness and public engagement.

The results above show that three sub-dimensions of genuineness, namely “genuine response”, “functional interactivity” and “genuineness in Chinese culture”, have significant associations with public engagement on social media. Therefore, we intend to further examine the association between the sub-dimensions in the four genuineness dimensions and public engagement. Table 5 summarizes the negative binomial regression results on the sub-dimensions of “self-disclosure”, “genuine response”, “functional interactivity”, “genuineness in Chinese culture” and the number of shares, likes, comments and positive comments.

In response to RQ2a, we found that in the dimension of “self-discourse”, “disclosure of personal life” had positive effects on the number of user shares and likes, while “disclosure of personal expertise in pediatrics” is positively associated with number of shares. For every extra sentence in “disclosure of personal life” and “disclosure of personal expertise in pediatrics”, 1.23 (*p* = 0.003) and 1.13 (*p* = 0.005) times more shares were generated. For every extra sentence in “disclosure of personal life”, 1.26 times (*p* = 0.0003) more likes were expected. However, the “disclosure of personal thoughts and feelings” was negatively associated with the number of comments. For every extra sentence in “disclosure of personal thoughts and feelings”, 0.89 times (*p* = 0.033) fewer comments were expected (See Table 5). 

Regarding the sub-dimensions in “genuine response” (RQ2b), our findings revealed that “consistency” had positive effect on the total number of likes, comments, and positive comments. 1.402 times more likes (*p* = 0.0003), 1.581 times more comments (*p* < 0.0001) and 1.347 times more positive comments (*p* = 0.001) were witnessed for every extra sentence on “consistency” provided. Similarly, the sub-dimension of “knowledge, skill, experience and treatment advice” was positively associated with the number of comments and positive comments. For each extra sentence on “knowledge, skill, experience and treatment advice”, 1.342 times more comments (*p* < 0.0001) and 1.166 times more positive comments (*p* = 0.013) were yielded. However, the sub-dimension of “facilitation of hopefulness” was negatively associated with the number of likes and shares. For every extra sentence on “facilitation of hopefulness”, 0.55 times fewer shares (*p* = 0.001) and 0.742 times fewer likes (*p* = 0.023) were generated, as presented in Table 5.

For the dimension of “functional interactivity” (RQ2c), the “generation of return visits and conservation of visitors” had positive effects on the total number of shares and likes, whereas “dialogic loop” had a negative association with the number of comments and positive comments. 

For every additional “conservation of visitors and the generation of return visits” included, the shares and likes increased by 43% (*p* = 0.001) and 22% (*p* = 0.018) respectively while comments and positive comments decreased by 84% (*p* = 0.005) and 88% (*p* = 0.015) for every extra feature of “dialogic loop” provided, as shown in Table 5. 

Last but not least, “honesty” in the dimension of “genuineness in Chinese culture” (RQ2d) had a positive association with the number of shares, likes, and positive comments in contrast to kindness, which showed no significant association. For every extra lexical indicator on “honesty”, 1.158 times more shares (*p* = 0.001), 1.106 times more likes (*p* = 0.0004), and 1.07 times more positive comments (*p* = 0.007) were generated, as presented in Table 5.

## 4. Discussion

Our results revealed that a variety of genuineness dimensions was employed by the SMIP to communicate with the public on social media. The findings yielded insights into how the “genuine response” alongside “functional interactivity” and “genuineness in Chinese culture” played an active role in engaging the public. 

### 4.1. Strong Effect of Genuine Response on Public Shares and Comments

Corroborated with previous studies [33,36,83], our findings revealed that responses with high level of consistency and expert knowledge were positively associated with public engagement (Table 5). A doctor responded by acknowledging the public’s need helps develop a trustful relationship [84], even in online doctor-public communication. Furthermore, response with medical knowledge and treatment advice indicates the doctor’s understanding of patient’s concerns and his/her intention to address the issues [85]. This could be the reasons attributing to the positive association between “genuine response” and the number of likes and comments, especially the positive comments.

“Genuine response” that aimed to address patients’ concerns created more opportunities for the public to express feelings (e.g., grateful, satisfied) in the comment threads, and allowed them to continually ask questions if their concerns were not fully addressed. The phrases “thanks, doctor,” “beneficial advice,” “learn a lot,” “really appreciate your patient guidance” and “what I need to do in the next step” were frequently unveiled under the comment threads. 

### 4.2. Interactive Features Promote Public Shares

In line with previous studies [37,40,42,57], our results also revealed the strong effect of “functional interactivity” on public shares (Table 4 and Table 5). We found a range of interactive features, in the form of links/hashtags, such as “#SIMP name+topic#”, “@+other online users name” and “link to other Weibo pages”, employed on the SMIP posts which foster the public’s access to various and detailed information. 

Hashtags lead users to daily hot topics where users can make synchronous conversations, discuss relevant issues with others, and share insightful ideas [57]. Links enable users to return the site and increase the time of stay when reading messages [42]. Owing to word limit on Weibo, the SMIP messages may not explain the ins and outs of a health problem thoroughly. The offering of external links expands messages in greater detail and strengthens the usefulness of corresponding posts, thereby fostering information sharing. Given that the act of sharing can potentially reach out to a large audience, online doctors can adopt interactive elements to express genuineness and extend their influences.

### 4.3. Honesty is Highly Valued in Chinese Culture

Noticeably, the sub-dimension “honesty” positively engendered public engagement of likes, shares and positive comments (Table 5). The expressions in honesty mainly involve verbs and adverbs related to explanation, personal views, and facts, such as “for instance,” “include,” “I think,” “I suggest,” “according to,” and “the document shows.” Tuckett [86] specified that “honesty” is “perceived as truth-telling” (p. 500), and the extent to which truth-telling is preferred is highly related to culture and context. As noted in previous study [86], “honesty” is a fundamentally ethical principle in doctor-patient relationships. The majority of patients in China demonstrate that they want truthfulness and authenticity about their illness, which could enable them to manage uncertainty and make decisions independently [50]. This might also explain the negative association between sub-dimension of “facilitation of hopefulness” in the “genuine response” and shares and likes. To some extent, expression of hopefulness is intended to comfort patients instead of telling the whole truth [33,36], and the truthfulness of such expressions often arises suspicion.

Also, the shared post represents the user [62]. A previous study [87] found that online self-presentation was a crucial part of impression management, in which the public carefully evaluated someone by how he presented himself. This suggests that sharing requires more cognitive effort [64]. Given that “honesty is the traditional morality of Chinese nationalities and is regarded as the basis of the making of a man” ([88], p. 177), it is not surprising to see the public’s willingness in sharing “honest” so as to promote positive personality traits on social media. 

### 4.4. Self-Disclosure as a Controversial Communication Behavior

Despite previous studies suggested doctor’s “self-disclosure” may have a positive impact on patients’ reactions and foster a stronger therapeutic relationship, our results reveal that “self-disclosure” has no significant association with any level of public engagement (Table 4). Beach et al. [89] argued that doctors’ personal disclosure to patients have sometimes been regarded as a boundary transgression. Doctors should be more careful about disclosing personal information [86]. “Self-disclosure” has been viewed as a positive intervention in doctor-patient communication but it could also hinder effective communication and lead to negativity [90]. Kelly and Achter [91] found that patients concerned the helpfulness and benefits of the disclosure information from doctors for their decision on engagement. If they think the information would be useful for their situations, they are willing to further interact with doctors and listen to their suggestions [92]. However, given that “self-disclosure” messages in this study mainly involve personal life, opinions, and feelings that may not be relevant to the public’s concerns and problems, a lower level of public engagement is expected. Forest and Wood [90] commented that it is not surprising that people may disapprove or doubt the information provided by therapists who share personal opinions and experience frequently. Likewise, disclosure of personal expertise may involve the discouraging expressions [92], which makes the public feel sad and stressful. In addition, McDaniel et al. [93] found that the frequent statements about the doctor’s personal life (e.g., family, habit) and professional information are, occasionally, of little value to impair the doctor-patient relationship because they may result in fewer opportunities for patients to express themselves. General information is prevalent in SMIP’s posts on Weibo as each post is limited to 140 words, but there are a variety of followers with different needs. In other words, the posts cannot meet everyone’s demands even though SMIP want to provide detailed information. Therefore, the public may not react to some information that is not tailored to their problems.

## 5. Conclusions

Academically, this study contributes to the research of health communication in the following aspects: (1) developed an integrated framework to conceptualize and measure genuineness in social media communication and (2) shed lights in the understanding of effect of genuineness on Chinese public engagement in SMIP online communication. 

In terms of practical implications, this study provides insights to health information providers such as pediatricians in engaging public on social media communication. For instance, the use of “genuine response” could raise public awareness which in turn facilitates the fostering of a healthy lifestyle. 

This study also has strong social implications. In recent years, the Chinese government has placed public health at the center of the country’s entire policy-making agenda and initiated a national long-term strategic public health plan. One of the missions is to improve the well-being of citizens by spreading health knowledge and promoting quality medical care. Sustainable investment and efforts have been put into relevant fields, especially online, because in China, the Internet is becoming increasingly important in health care and offers a great number of platforms for patients to seek health information and conduct medical consultations. Therefore, this research provides insights into the formation of high-quality health information and productive medical services.

Given the ongoing interaction between the public and SMIP on Weibo, further scrutiny on SMIP communication is highly warranted. Future studies could expand the database and include multiple social media outlets such as Wechat, TouTiao, and Baidu to fully examine the associations between different dimensions of genuineness and public engagement. With regard to the coding procedure, the data were not exhaustively coded as double coding within dimension was not allowed in this study. Lastly,“genuineness in Chinese culture”in this study was measured based on the lexical markers derived from the Chinese dictionary and relevant studies. To ensure a higher level of objectivity, we could harvest the most used words on content and style of genuineness by interviewing/surveying social media users that have interacted with the SMIP on Weibo.

## Figures and Tables

**Table 1:** Top 10 SMIP in Sina Weibo and the total number of their postings and followers.

Rank	Name of the SMIP (English Translation)	V label in Sina Webo	Total No. of Posts (Mar 1 to Aug 31, 2019)	Average No. of Posts/Day	Followers (as of Mar 1, 2019)
1	崔玉濤 (Cui Yu Tao)	Pediatrician	368	2	7,700,000
2	蝦米媽咪 (Xia Mi Mommy)	Pediatrician	430	2.337	4,870,000
3	鮑秀蘭診室 (Bao Xiulan clinic)	Pediatrician	5160	28.044	4,630,000
4	張思萊醫師 (Physician Zhang Silai)	Pediatrician	12,915	70.19	3,330,000
5	小兒外科裴醫生 (Pediatric Surgeon Doctor Pei)	Pediatrician	759	4.125	1,840,000
6	醫生媽媽歐茜 (Doctor Mommy Ou Qie)	Pediatrician	688	3.739	1,720,000
7	兒童營養師劉長偉 (Child nutritionist Liu Changwei)	Pediatrician	523	2.842	1,330,000
8	兒科醫生雨滴 (Pediatrician Yu Di)	Pediatrician	2661	14.462	990,000
9	兒科醫生魚小南 (Pediatrician Yu Xiaonan)	Pediatrician	436	2.37	930,000
10	張亞停醫生 (Doctor Zhang Yating)	Pediatrician	1580	8.587	890,000

**Table 2:** The major dimensions, sub-dimensions and descriptors of the code book.

Dimensions	Sub-Dimensions	Descriptors
Self-disclosure	Disclosure of personal life	Share daily life (e.g., habit, activity, behavior)
	Disclosure of personal thoughts and feelings	Uncover the views and attitudes towards something
	Disclosure of personal expertise in pediatrics	Share the content related to pediatrics and children’s health
Genuine response	Consistency	Rephrase or repeat public’s questions and expressions to ensure their concerns are well understood
	Knowledge, skill, experience and treatment advice	Provide treatment advice with elaborations for public concerns; Provide medical knowledge and experience to raise public health awareness
	Facilitation of hopefulness	Express a sense of hope, e.g., “you can handle that”, “no worries”, “I can help you”
Functional interactivity	Generation of return visits and conservation of visitors	Provide links/hashtags to the pediatrician’s clinic/organization/own Weibo page; Provide links/hashtags to other social networks in which the Pediatrician is present; Provide links/hashtags to other Weibo pages
	Dialogic loop	Reply by the pediatrician to a user’s comment on a post/comment thread; Multimedia (type: text, live chat, video, audio); Encourage more enquiry; Ask questions
Genuineness in Chinese culture	Honesty	Lexical indicators related to reasoning and explanation, e.g., because, so; Lexical indicators related to personal sharing and views, e.g., I think, I contend, I prefer; Lexical indicators related to truth/facts, e.g., In fact, the truth is, the evidence reveals
	Kindness	Expressions of care, e.g., is that okay, are you satisfied, is this clear for you; Expressions of friendliness, e.g., hello, could you please, welcome; Expressions of gratitude, e.g., thanks, appreciate it; Expressions of blessing, e.g., wish you, no worries, everything will be fine; Expressions of compliments, e.g., good question, you are right.

**Table 3:** Summary of Inter-Rater Reliability Statistics.

Dimensions/Public Response	Sub-Dimensions	Percent Agreement	Scott’s Pi	Cohen’s Kappa	Krippendorff’s Alpha
Self-disclosure	Disclosure of personal life	95%	0.91	0.91	0.912
	Disclosure of personal thoughts and feelings	96.7%	0.969	0.969	0.97
	Disclosure of personal expertise in pediatrics	98.3%	0.807	0.807	0.808
Genuine response	Consistency	91.7%	0.896	0.896	0.897
	Knowledge, skill, experience and treatment advice	95%	0.845	0.845	0.846
	Facilitation of hopefulness	95%	0.923	0.923	0.923
Functional interactivity	Generation of return visits and conservation of visitors	96.7%	0.928	0.928	0.929
	Dialogic loop	93.3%	0.867	0.867	0.868
Genuineness in Chinese culture	Honesty	96.7%	0.934	0.934	0.935
	Kindness	100%	1	1	1
Public response	Shares	100%	1	1	1
	Likes	100%	1	1	1
	Comments	100%	1	1	1
	Positive comments	93.3%	0.867	0.867	0.868

**Table 4:** Negative Binomial Regression Results on the Four Dimensions of Genuineness and the Number of Shares, Likes, Comments and Positive Comments.

Dimensions	Number of Shares			Number of Likes			Number of Comments			Number of Positive Comments		
	B	SE	Exp(b)	B	SE	Exp(b)	B	SE	Exp(b)	B	SE	Exp(b)
Self-disclosure	−0.037	0.05	0.964	0.029	0.05	1.030	0.068	0.04	1.070	0.014	0.03	1.014
Genuine response	−0.247 ***	0.05	0.782	0.038	0.05	1.039	0.296 ****	0.04	1.344	0.148 ***	0.04	1.160
Functional interactivity	0.180 ****	0.05	1.198	0.015	0.04	1.015	−0.158 ***	0.04	0.854	−0.090 *	0.04	0.914
Genuineness in Chinese culture	0.115 **	0.04	1.122	0.064	0.04	1.067	−0.007	0.03	0.993	0.052	0.03	1.054

*p* < 0.05 *, *p* < 0.01 **, *p* < 0.001 ***, *p* < 0.0001 ****.

**Table 5:** Negative Binomial Regression Results on the Sub-dimensions of “Self-disclosure”, “Genuine response”, “Functional interactivity”, “Genuineness in Chinese Culture” and the Number of Shares, Likes, Comments and Positive Comments.

Dimensions	Number of Shares			Number of Likes			Number of Comments			Number of Positive Comments		
	B	SE	Exp(b)	B	SE	Exp(b)	B	SE	Exp(b)	B	SE	Exp(b)
**Self-disclosure**												
Disclosure of personal life	0.209 **	0.07	1.232	0.229 ***	0.06	1.257	−0.083	0.07	0.921	0.055	0.07	1.057
Disclosure of personal thoughts and feelings	−0.068	0.09	0.935	−0.016	0.08	0.984	−0.116 *	0.06	0.890	−0.084	0.07	0.919
Disclosure of personal expertise in pediatrics	0.125 **	0.04	1.133	0.017	0.03	1.017	−0.036	0.03	0.965	0.003	0.03	1.002
**Genuine response**												
Consistency	−0.189	0.12	0.828	0.338 ***	0.09	1.402	0.458 ****	0.10	1.581	0.298 ***	0.09	1.347
Knowledge, skills, experience and treatment advice	−0.145	0.08	0.865	0.039	0.06	1.039	0.294 ****	0.07	1.342	0.153 *	0.06	1.166
Facilitation of hopefulness	−0.598 ***	0.18	0.550	−0.298 *	0.13	0.742	0.035	0.15	1.036	−0.073	0.13	0.929
**Functional interactivity**												
Conservation of visitors and generation of return visits	0.354 ***	0.10	1.425	0.197 *	0.08	1.218	−0.194 *	0.08	0.824	−0.032	0.07	0.969
Dialogic loop	0.078	0.07	1.018	−0.004	0.05	0.996	−0.170 **	0.06	0.844	−0.132 *	0.055	0.876
**Genuineness in Chinese culture**												
Honesty	0.147 ***	0.05	1.158	0.100 ***	0.03	1.106	0.039	0.04	1.039	0.068 **	0.03	1.070
Kindness	0.047	0.12	1.048	−0.005	0.09	0.995	−0.119	0.09	0.888	0.053	0.07	1.055

*p* < 0.05 *, *p* < 0.01 **, *p* < 0.001 ***, *p* < 0.0001 ****.

## Appendix A

Exemplification of four genuineness dimensions and relevant expression identified in the corpus.

Coding items	Examples
Self-disclosure	
1. Disclosure of personal life	No. 7: May.12, 20:36 上午带小七一起去仙林金鹰广场观看全民营养周启动仪式，仪式活动很棒，小七表现也很棒，天很热，小七全程很认真地看完。午餐时饿了，吃哈密瓜，鸡翅，意大利面，还吃了半块含有牛奶和鸡蛋甜点，本来知道她会过敏，不给她吃，抢着要吃，不过吃了没有出现明显过敏症状。 Literal translation: In the morning, I took Xiaoqi to Xianlin Golden Eagle Square to watch the launching ceremony of national nutrition week. The ceremony was great. Xiaoqi performed well. It was scorching, but Xiaoqi watched the whole process carefully. I ate cantaloupe, chicken wings, spaghetti, and half of the dessert at lunch. I knew that she would be allergic, so I did not give the food to her. However, she insisted on eating them, and I had not found any obvious allergic symptoms with her after eating.
2. Disclosure of personal thoughts and feelings	No. 5: Aug.4, 19:36 谢谢信任和认可。不过我们作为管理者知道，目前还是有蛮多可以改进和提高的地方。 Literal translation: Thank you for your trust and recognition. However, as a manager, we know that there are still many things that can be improved.
3. Disclosure of personal expertise in pediatrics	No. 3 July.19, 13:02 很多宝宝都会有哭闹的现象，但是有些宝宝的哭闹让爸爸妈妈很不省心，甚至有点心力交瘁。如果他会比其他宝宝都爱哭，而且哭的时间还很长，这个时候家长就要警惕宝宝是不是肠绞痛。 Literal translation: Many babies have crying phenomenon, which makes parents uneasy and exhausted. If he cries more than other babies, and the crying time is very long, parents should be wary of colic problem.
Genuine response	
1. Consistency	
Rephrase public’s question and concern	No 1: Mar.10, 06:00 User one: 你好医生, 宝宝9个月，能逗笑，会发妈妈的音，能抓玩具，能扶着腋窝站立，不能独坐超过7.8秒，要东倒西歪的，趴几秒也要哭就把两只手放两边，请问这种情况做康复能好吗？ Literal translation: Hello doctor, my baby is 9 months old. He can laugh, make the voice of “mommy”, grasp toys, and stand by armpit, but can not sit alone for more than 7 or 8 seconds. He often comes to cry for a few seconds when he is prostrating. I wonder whether he would recover from this situation? Pediatrician: “9个月，能逗笑，会发妈妈音，能抓玩具，能扶着腋窝站立，不能独坐超过7.8秒，趴几秒也要哭就把两只手放两边。”据叙述，大运动发育落后。“能扶着腋窝站立”这一定是家长扶着孩子站，不利于大运动发育，反而会有消极作用。建议看神经康复科医生，是发育问题，还是家长养育问题。 Literal translation: *“Nine months, can make you laugh, can make your mother’s voice, can grasp toys, can stand by your armpit, cannot sit alone for more than 7 or 8 seconds, can cry even if you lie down for a few seconds.”* According to your illustration, it could be said that the development of large motor skills is backward. *“Can support the armpit to stand”* indicates parents was helping the child to stand, which is not conducive to the development of large motor skills and will have a negative effect. I suggest you should consult a neurologist to see if it is a developmental or a parenting problem.
2. Knowledge, skill, experience and treatment advice	
Provide treatment advice to address public’s concern	No 2: May.9, 23:11 User one: 您好 吖一岁15天 不吃奶瓶已经十天了 只吃亲喂 奶量肯定不够 什么缘故？怎么办呢？ Literal translation: Hello, my son is one year and 15 days old and has not fed by bottle for 10 days. I think the amount of breast-feeding may not be enough. What is the reason and what shall I do? Pediatrician: 可以杯子喂奶 同时试试奶酪和酸奶。 Literal translation: You can try to feed him by a cup. At the same time, feed him with some cheeses and yogurt.
Provide medical knowledge and experience to raise public’s health awareness	No. 1: Mar.18, 06:23 User one: 崔医生，哺乳期可以烫头发染头发吗？ Literal translation: Dr Cui, can I have a perm and dye my hair during lactation? Pediatrician: 只要妈妈烫头、染头后没有不适，就不会通过乳汁影响孩子，从而造成不适。只是不要让孩子舔妈妈的头发而已。爱美之心，人皆有之。生完宝宝后，绝大多数妈妈都想尽快恢复体型，皮肤光鲜亮丽等。为此染发，烫头，染指甲等都可以进行。 妈妈会自己掌握分寸的。 Literal translation: As long as the mother has no discomfort after perming and dyeing her hair, she would not affect her children through breastfeeding. Just keep in mind that do not let the child lick his/her mother’s hair. Everyone has a desire for beauty. After giving birth to the baby, most mothers want to recover their body shape and bright skin as soon as possible. Thus, hair dyeing, perm, fingernail dyeing can be carried out. Mothers will take care of themselves.
3. Facilitation of hopefulness	No.10: July.26, 06:30 User one: 我儿子8个月出2个牙，到一周了去体检，人家8个牙我们6个牙。1岁3个月才8个牙，现在1岁半了8个长出来完的还有4个没长出来完，每天都有吃钙吃鱼肝油呢不知道怎回事？ Literal translation: My son has 2 teeth within 8 months. When he was one year and 3 months old, he only had 8 teeth; At present, he is one and a half years old but only with 4 teeth growing. We eat the food, e.g., cod liver oil filled with calcium everyday, so what is wrong? Pediatrician: 只要有牙齿出就说明牙齿发育没有问题，耐心等待即可。放轻松。 Literal translation: As long as you can see teeth out and growing, there is no problem with tooth development. Just be patient and relax.
Functional Interactivity	
1. Conservation of visitors and Generation of return visits	
Link to the Pediatrician’s clinic/organization/own Weibo page	No.3: Mar.31, 12:05 哺乳期妈妈生病就要扛吗？还可以喂奶吗？很多妈妈在哺乳期的时候，十分谨慎，生怕自己生病后不能哺乳，从而影响宝宝生长。有的则是因为家里老人怪自己生病，怕传染给孩子。这个问题，点击“*《我的诊室》*”了解更多，网页链接 Literal translation: Is it necessary for lactation mothers to endure illness with silence? Can they still provide breastfeeding? Many mothers fear that they may not be able to breastfeed when they are ill in case of affecting the growth of their babies. Some concern that the child’s grandparents will blame them for being sick and infecting their children. For this concern, click *“my clinic”* to learn more
Link to other social networks in which the Pediatrician is present	No.1: Apr.21, 06:22 孩子出现喂养不适很可能与疫苗有关，但不应该是大问题。如果孩子没有新的不适，家长耐心等待，1-2周会自然恢复。还要关注排便情况。#崔玉涛讲疫苗#。 Literal translation: Feeding discomfort in children is likely to be related to vaccines, but it is not a big problem. If the child has not emerged new discomfort, the parents need to wait patiently. The child will recover naturally in 1-2 weeks. Also, you should pay attention to defecation. #Cui Yutao talks about vaccines#.
Link to other Weibo pages	No.2: May.19, 15:03 #贵州省龙江村# #贵州省黄岗村#【从一次关注开始，我和贵州侗寨孩子们的故事】所有认定的事情都贵在坚持。 Literal translation: #Longjiang village in Guizhou Province# #Huanggang village in Guizhou Province# <the first story of my experience with Dongzhai children >, all the things we are insisting on are worthwhile.
2. Dialogic loop	
Reply by the Pediatrician to a user’s comment in a post/comment thread	No.8: June.26, 14:33 @pediatrician: 哈哈，太可爱了//@User：我闺女也这样，笑得我呀！ Literal translation: @pediatrician: ha ha, it is so cute//@User: my daughter was just like this. How amusing!
Multimedia (type: text, live chat, video, audio)	No 10: Aug.26, 11:46
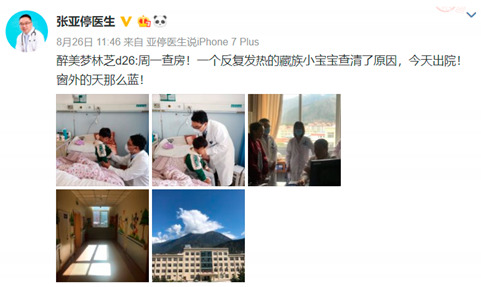
Encourage more enquiry	No 2: June.9, 09:39 User one: 关于图九有点疑问，小宝宝不是需要晒太阳补充VD吗？如果十点之前也需要穿长袖长裤、在阴凉处防晒？ Literal translation: There is one question about figure 9. Isn’t it necessary for the baby to supplement VD by basking in the sun? I just wonder if we need to wear long sleeved trousers and sunscreen in the shade before 10 o’clock? Pediatrician: 还是建议补充维生素D 具体原因可以在我微博搜索关键词 维生素D Literal translation: I recommend children should supplement vitamin D. The specific reason can be found through searching keyword: “Vitamin D” on my Weibo account.
Ask questions	No. 2: Mar.19, 23:08 儿童白血病是不治之症吗？身体里的血液细胞们怎么了？得了白血病可能会有哪些症状，要做哪些检查，有哪些治疗方案……请花7分钟了解儿童白血病。 Literal translation: Is leukemia in children an incurable disease? What happened to the blood cells in the body? What are the symptoms of leukemia, what tests are needed to do and what treatment options are available... Please take 7 minutes to learn about children leukemia.
Genuineness in Chinese culture	
1. Honesty	
Lexical indicators related to reasoning and explanation	No.6: Mar.14, 20:46 但你可能想不到的是，全球最优秀的高校，排在前列的，比如哈佛、MIT、耶鲁、哥大，都是私立学校。推广到医疗领域，全美最优秀的医疗机构，比如梅奥诊所、克利夫兰医学中心、约翰霍普金斯医院，也都不是公立医院。 Literal translation: But what you have not known is that the best universities in the world are mainly private colleges, such as Harvard, MIT, Yale, and Columbia. As for the medical field, the best medical institutions in the United States are also not public hospitals, such as Mayo Clinic, Cleveland Medical Center, and Johns Hopkins Hospital.
Lexical indicators related to personal sharing and views	No 1: June.17, 06:21 看检测不是过敏原检测，而是食物不耐受检测，不能依此诊断牛奶过敏，更谈不上“中度过敏”。我认为没必要检测过敏原，因为过敏原检测也只针对IgE介导过敏，不是全部。 Literal translation: The check is not an allergen test but a food intolerance test. Thus it is not possible to diagnose milk allergy, letting alone “moderate allergy.” I do not think it is necessary to detect allergens because allergen testing only targets IgE-mediated allergies, not all of types.
Lexical indicators related to truth/facts	No 8: July.28, 13:15 专家研究表明，从卫生经济学角度来说，儿童早期的投入是生命全周期中人类资本投入产出比最高的，早期的投资回报率达到1：7以上，因此儿童早期潜能的开发不仅决定了个体的发展潜力，同时也深刻影响着我们国家人力资源的竞争力。 Literal translation: Experts research shows that from the perspective of health economics, the early childhood investment is the highest ratio of human capital input to output in the whole life cycle. The early return on investment is more than 1:7, so the development of early childhood potential not only determines the development potential of individuals but also profoundly affects the competitiveness of our country’s human resources.
2. Kindness	
Expression of care	No.6: Mar.26, 08:57 如果只能推荐一本育儿书，那一定是《美国儿科学会育儿百科》，携手丁香妈妈 开发了这套系统性育儿课，希望帮你在纷繁复杂的信息中，筛选出科学、权威、全面的育儿知识，并且把它以一种比书本更加轻松的方式讲给你听。这并不容易的育儿路，我们愿意陪着你一起走。 Literal translation: If I can recommend a parenting book, it must be 《the American Academy of Pediatrics Parenting Encyclopedia》. Together with Clove Mama, this systematic parenting class has been developed. I hope to help you screen out scientific, authoritative and comprehensive knowledge in the complicated information, and tell them to you in a more natural way. This is not easy but we are willing to accompany with you.
Expression of friendliness	No 8: Apr.7, 21:18 我是您身边的医生朋友雨滴，如果觉得对你有帮助，欢迎帮我留言转发点赞。 Literal translation: I am a doctor and friend around you. If you feel it is helpful to you, welcome to help me forward my message.
Expression of gratitude	No 7. June.16, 15:35 感谢！<玫瑰> <玫瑰> Literal translation: Thanks! <rose> <rose>
Expression of blessing	No 8. Mar. 25, 15:33 愿所有的孩子平安幸福，希望天下所有的父母都能看到。 Literal translation: May all children be safe and happy, and hope that all parents in the world can see it.
Expression of compliment	No 1. Apr. 9, 06:13 User one: 那需不需要给孩子补充维生素k呢? Literal translation: Do we need vitamin K for the children? Pediatrician: 问的很好！首先要保证孩子青菜等深色蔬菜的摄入。青菜含有维生素K，在肠道正常菌群作用下，产生维生素K1和K2。不光是孩子，成人也是如此，进食绿叶菜好处多多。现在已有维生素D3和维生素K2的混合制剂了。 Literal translation: Good question! First of all, we should ensure the intake of vegetables for the kids. Vegetables contain vitamin K, which is produced by healthy intestinal flora. Eating green leafy vegetables has many advantages for both children and adults. There is a mixture of vitamin D3 and vitamin K2 for now.
